# Full-color, large area, transmissive holograms enabled by multi-level diffractive optics

**DOI:** 10.1038/s41598-017-06229-5

**Published:** 2017-07-19

**Authors:** Nabil Mohammad, Monjurul Meem, Xiaowen Wan, Rajesh Menon

**Affiliations:** Department of Electrical and Computer Engineering, University of Utah, Salt Lake City, UT 84112 USA

## Abstract

We show that multi-level diffractive microstructures can enable broadband, on-axis transmissive holograms that can project complex full-color images, which are invariant to viewing angle. Compared to alternatives like metaholograms, diffractive holograms utilize much larger minimum features (>10 µm), much smaller aspect ratios (<0.2) and thereby, can be fabricated in a single lithography step over relatively large areas (>30 mm ×30 mm). We designed, fabricated and characterized holograms that encode various full-color images. Our devices demonstrate absolute transmission efficiencies of >86% across the visible spectrum from 405 nm to 633 nm (peak value of about 92%), and excellent color fidelity. Furthermore, these devices do not exhibit polarization dependence. Finally, we emphasize that our devices exhibit negligible absorption and are phase-only holograms with high diffraction efficiency.

## Introduction

The word, hologram originates from the Greek word, holos, which means whole^1^. In general, the whole refers to the ability to control phase and amplitude of a wavefront to create a desired intensity image projection. In conventional holography, this is achieved via the interference between two coherent beams, one containing the information about the scene and another a reference beam^2^. Digital holograms and computer-generated holograms have also been used extensively to achieve the same effect either via spatial-light modulators^3^ or using surface-relief structures^4^. Lipmann photographs is a class of color holograms, where broadband interference fringes are recorded in a special photopolymer^5^. However, their efficiency and field of view are highly limited due to the underlying Bragg diffraction. It is well known that multi-level diffractive optics (also referred to as kinoforms) can achieve very high efficiencies at a single wavelength^6, 7^. However, extension to full color computer-generated holograms is challenging and typically requires one device for each color^8^. In addition, such surface-relief devices required multi-step lithographic processes, exhibited relatively low diffraction efficiencies and required coherent illumination. Metasurfaces, which may be defined as 2D photonic devices whose unit cells are comprised of sub-wavelength structures, have recently been applied to holography^9–15^. These devices can engineer the amplitude, phase and polarization of light. The key functional difference between conventional holograms and metasurfaces is the fact that metasurfaces can manipulate vector properties of the electromagnetic wave, namely polarization^16, 17^. Here, we emphasize that if one is interested in only the scalar properties of light, such as intensity images, then metasurfaces are *not* required. In fact, metasurface-based holograms require very complex fabrication due to the subwavelength constituent features. Furthermore, they generally suffer from polarization dependence and relatively small operating bandwidths. Previously, we have described numerical studies of broadband transmissive holograms with peak efficiency greater than 90% that do not share any of the disadvantages of metasurfaces^18^. Here, we experimentally demonstrate high-efficiency, on-axis, transparent, full-color holograms using such multi-level diffractive optics.

Broadband diffractive optical elements using multi-level super-wavelength features have been applied for spectrum-splitting and concentration in photovoltaics^19–21^, and for super-achromatic cylindrical lenses^22^. In this work, we extend the application of this concept to broadband computer-generated holography by designing, fabricating and characterizing a variety of holograms. We show that average transmission efficiencies of over 86% can be achieved experimentally for the visible spectrum (405 nm to 633 nm). Note that transmission efficiency is the figure of merit used to characterize holograms based on metasurfaces^9^. We further show that complex image projections with large viewing angles such as color photographs can be achieved. All our devices utilize minimum feature size of 10 μm or larger and can be readily manufactured using micro-imprinting or embossing techniques, potentially over large areas, if desired^23^.

**Figure 1:**
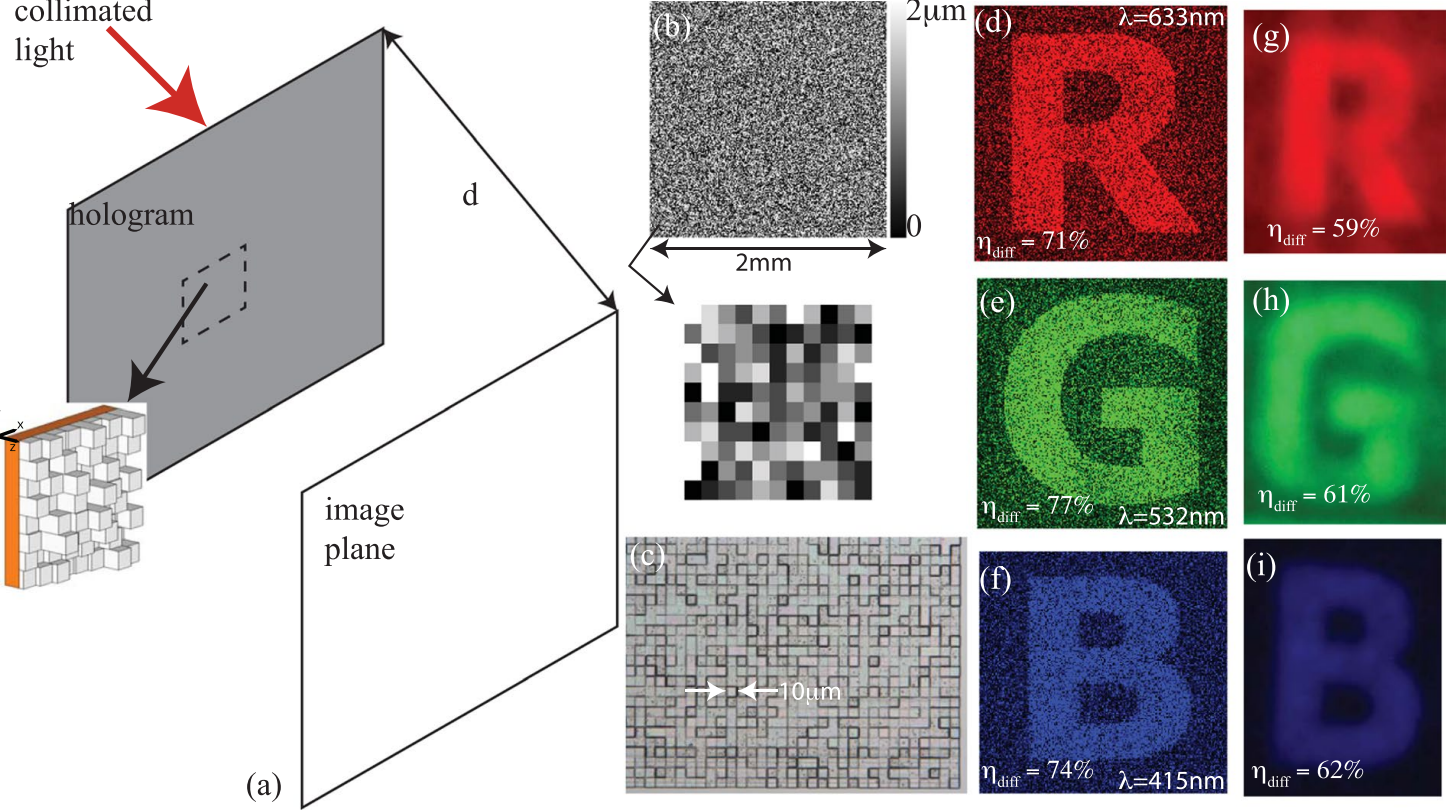
(**a**) Schematic of hologram. (**b**) Designed height map of color-encoded hologram. Bottom Inset: Magnified view of 11X10 pixels of bottom left corner. (**c**) Magnified optical micrograph of a small portion of the device. Simulated images at (**d**) *λ* = 633 nm, (**e**) *λ* = 532 nm and (**f**) *λ* = 415 nm are shown. The corresponding experimental images reflecting of a white opaque screen are shown in (**g**)–(**i**), respectively. The simulated and measured diffraction efficiencies are noted in each image.

Each hologram is comprised of square pixels as shown in Fig. 1(a). Each pixel has a width, Δ and the heights of the pixels can vary from 0 to a maximum height of H in steps of Δh. When illuminated with appropriate wavelenghts of light, the hologram produces an image at a certain distance, d. The pixel heights are selected using an optimization procedure based on the target design as described below. In this work, we used 3 different target images: a color-encoded image, where each color produces a different image (overlapping in space), a Macbeth color-chart to showcase the range of colors, and finally, a color photograph of an outdoor scene. In the first design, our minimum feature size is 10 μm and maximum pixel height is 2 μm. In the 2^nd^ design, the minimum feature size is 20 μm and the maximum pixel height is 2 μm, while for the 3^rd^ design, the minimum feature size is 20 μm and the maximum pixel height is 2.4 μm. The large pixel widths ensure that these device are polarization independent. In all cases, we designed the devices for 3 discrete wavelengths, 405 nm, 532 nm and 633 nm. All the devices were designed using periodic boundary conditions. Other geometric and design parameters are fully described in the supplementary information.

The holograms are designed using nonlinear optimization with the objective of maximizing a figure of a merit defined as^18^:

1 $$\eta =\frac{1}{N}\sum _{\lambda }\frac{{{\rm{\Sigma }}}_{m}{{\rm{\Sigma }}}_{n}{I}_{T}^{(\lambda )}|U({p}_{m,n}){|}^{2}}{{P}_{in}^{(\lambda )}},$$

where, *η* is the wavelength averaged diffraction efficiency, *λ* is the design wavelength, *N* is the number of design wavelengths, ${I}_{T}^{(\lambda )}$ is the target image pattern at wavelength *λ*, *U*(*p*_*m*,*n*_) is the complex amplitude at the reconstruction plane diffracted by the hologram with height profile distribution *p*_*m*,*n*_, *m* and *n* are the pixel indisces, and ${P}_{in}^{(\lambda )}$ is the input power at wavelength *λ*. The objective of the optimization is to determine a height profile (*p*_*m*,*n*_) so that the wavelength averaged diffraction efficiency is maximized.

The holograms were fabricated using single-step grayscale lithography on a glass wafer spin coated with positive photoresist. In order to emulate the periodic boundary conditions used during design, the same design was repeated 3 times in the X direction and 3 times in the Y direction during fabrication. Details of the fabrication process are described in the supplementary information. For characterization, each hologram was illuminated by a collimated beam from a supercontinuum source with a tunable bandpass filter or from a white collimated backlight. The projected intensity images were captured either onto an image sensor (see Fig. S1) or projected onto a screen and photographed (see Fig. S2). A white translucent screen was used to capture on-axis images in transmission, while an opaque white screen was used to capture off-axis images in reflection. Further details of the imaging setup and characterization procedure are described in section 3 of the supplementary information.

The diffraction efficiency (η_diff_)was calculated as the power inside the outline of the target image divided by the total power inside the aperture of the hologram (see equation 1). This definition follows that used in ref. 10 and we adopt this same metric during design. This metric was measured only for the simple images in the color-encoded hologram. For all holograms, we also measured the transmission efficiency (η_trans_) as described later. Details of the efficiency measurements are included in the supplementary information.

The target images for the color-encoded hologram are the letters “R”, “G” and “B” at *λ* = 633 nm, 532 nm and 405 nm, respectively. The designed pixel-height distribution is shown in Fig. 1(b), while the optical micrograph of a small portion of the device is shown in Fig. 1(c). We measured the pixel heights of the fabricated device and estimated the average error as only 46 nm (see section 5 of the supplementary information). Although the blue wavelength used for design was 405 nm, we had to use 415 nm during the experiments, since that was the lowest wavelength with sufficient power accessible with our super-continuum source. Furthermore, the quantum efficiency of the image sensor is low at 405 nm. The simulated image at 405 nm is included in the supplementary information (Fig. S4). The simulated images at *λ* = 633 nm, 532 nm and 415 nm are shown in Fig. 1(d)–(f), respectively. Photographs of the corresponding images reflected off an opaque white screen are shown in Fig. 1(g)–(i) (see Fig. S2(b) for setup). The illumination bandwidth in each case was 10 nm. Corresponding images were also captured directly onto an image sensor (Fig. S6 and setup in Fig. S1) and the diffraction efficiencies were computed from these images. There is good qualitative agreement between the measured and simulated images. The measured average diffraction efficiencies are lower than expected (61% in experiments vs 74% in simulations). The discrepancy can be partly attributed to imperfect collimation of the incident light, which also accounts for blurring of the image edges compared to the simulations. We also performed a careful experimental analysis of the spectral response of this hologram and described the results in Fig. S7. These images clearly show the transitions between the images at wavelengths intermmediate to the design wavelengths.

**Figure 2:**
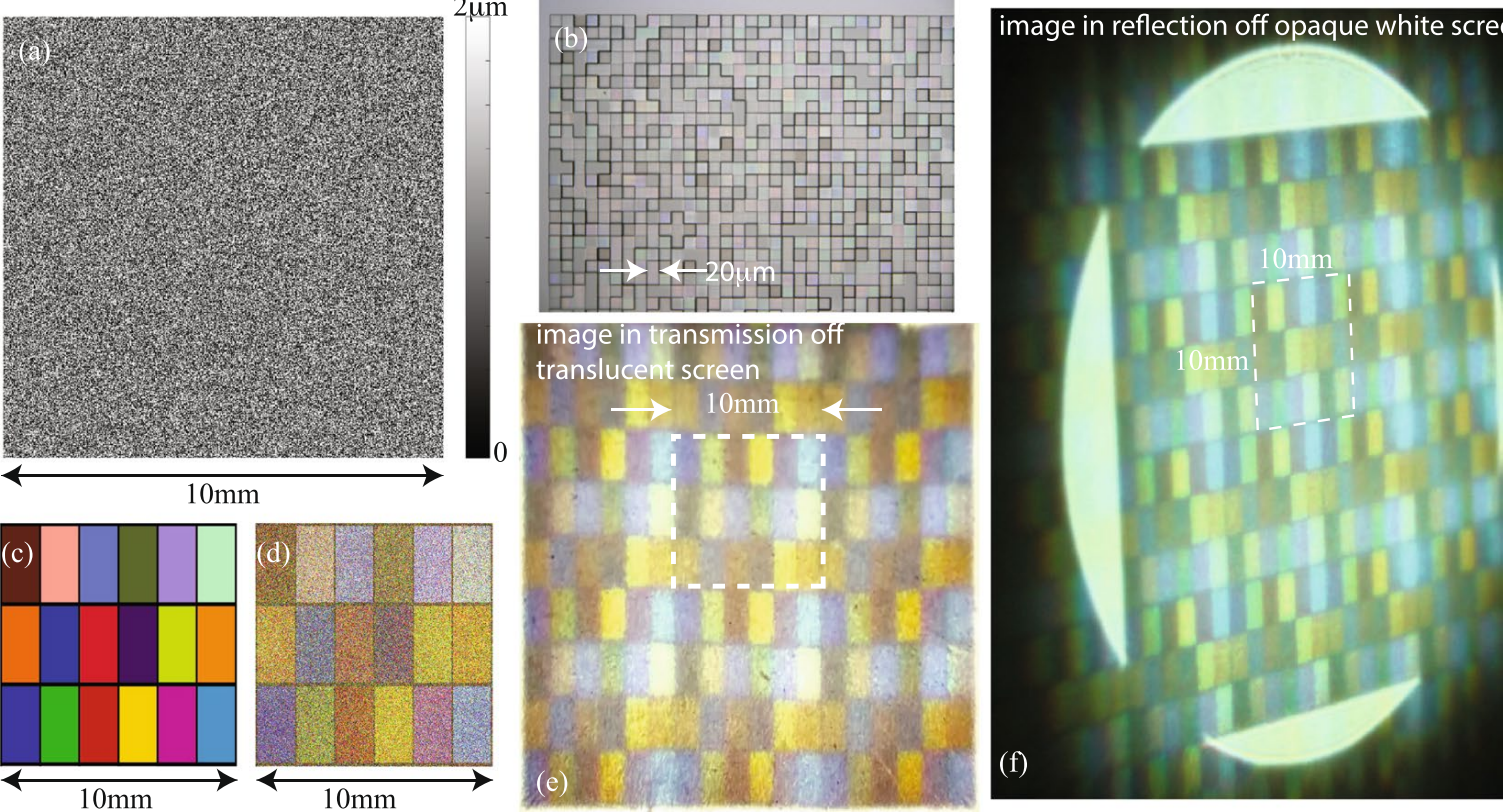
Hologram encoding a Macbeth color chart. (**a**) Design height map of the hologram. (**b**) Optical micrograph of a portion of the hologram. (**c**) Target image of the Macbeth chart. (**d**) Simulated image created by the hologram, when it is illuminated by the design wavelengths. (**e**) Photograph of the image on a white translucent screen taken in transmission at normal viewing angle. (**f**) Photograph of the image reflected of a white opaque screen taken at an oblique viewing angle. Note that the color image does not change with viewing angle (also see Figs S10 and S11). Illumination is full white spectrum from super-continuum source for both (**e**) and (**f**).

The second device we designed had a target image of a portion of the Macbeth color chart. The design pixel-height distribution is shown in Fig. 2(a) and an optical micrograph of a small portion of the fabricated device is shown in Fig. 2(b). The target image, the simulated image and a photograph of the experimental image projected onto a translucent white screen (see Fig. S2(a) for details) are shown in Fig. 2(c),(d) and (e), respectively. The dashed white lines in Fig. 2(e) demarcate one period of the image. Figure 2(f) shows the photograph of the image projected onto an opaque white screen (see Fig. S2(b) for details). Note that the photograph was taken at an oblique viewing angle illustrating that the image quality is maintained for a large range of viewing angles. Photographs taken at many other viewing angles are also shown in Fig. S10. Full white spectrum from the super-continuum source (Fig. S3 blue curve) was used as illumination in both cases. The simulated diffraction efficiencies (from equation 1) for this device were 64%, 53% and 65% at *λ* = 633 nm, 532 nm and 405 nm, respectively. The color range of the Macbeth chart is reproduced reasonably well considering that the design was performed only for 3 discrete wavelengths. Using more wavelengths during design will increase the color-reproduction accuracy.

**Figure 3:**
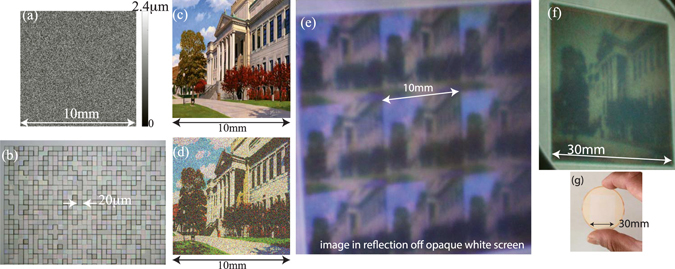
Hologram encoding a color photograph. (**a**) Design height map of the hologram. (**b**) Optical micrograph of a portion of the fabricated device. (**c**) Target image corresponding to the color photograph. Image courtesy of the University of Utah. (**d**) Simulated image created by the hologram, when it is illuminated by the design wavelengths. (**e**) Photograph of the image reflected of a white opaque screen taken at a small oblique viewing angle. (**f**) Photograph of image reflected of a white opaque screen for a single-period hologram containing 1500 X 1500 pixels in a single period. In both (**e**) and (**f**), the hologram is illuminated by the full visible band. (**g**) Photograph of single-period hologram. Its size is 30 mm X 30 mm.

The third device encodes a color photograph and its pixel-height distribution is shown in Fig. 3(a). An optical micrograph of a portion of the fabricated device is shown in Fig. 3(b). The simulated diffraction efficiencies were 77%, 85% and 86% for *λ* = 633 nm, 532 nm and 405 nm, respectively. The target image, the simulated image and a photograph of 3 X 3 periods of the projected image reflected off an opaque white screen are shown in Figs. 3(c), (d) and (e), respectively. Note that the photograph indicates good color reproduction even at an oblique viewing angle. Photographs at many more viewing angles are shown in Fig. S11. In order to increase the resolution of the projected image, we also designed and fabricated another device containing 1500 X 1500 pixels (Fig. S9). To reduce fabrication time, only one period of this device was fabricated and characterized. A photograph of the projected image reflecting off an opaque white screen is shown in Fig. 3(f). A photograph of this single-period hologram is shown in Fig. 3(g). This result coupled with the simulated image of a single-period hologram (Fig. S9) confirms that periodic boundary conditions are not a limitation of our design method. In all cases in Fig. 3, the devices were illuminated with the white collimated backlight.

**Figure 4:**
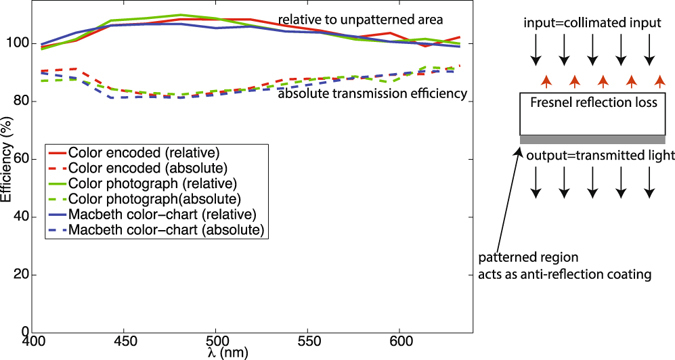
Measured transmission efficiency as a function of wavelength for the three hologram designs. The absolute transmission efficiencies are plotted using dashed lines, while the transmission efficiency relative to an unpatterned area is shown with solid lines. Right Inset shows how the patterned region reduces reflection losses, which allows for greater than 100% relative transmission.

We also measured the transmission efficiency of our devices. Note that transmission efficiency was used earlier as a figure of merit for metalens-based holograms^9^. First, an aperture of the same size as one period of the hologram was placed directly in front of the hologram (see Fig. S8). The absolute transmission efficiency was defined as the ratio of the power transmitted through the hologram to that incident on the hologram. We also measured the relative transmission efficiency, which is defined as the ratio of the power transmitted through the hologram to that transmitted through an unpatterned region. The results, as a function of illumination wavelength are plotted in Fig. 4 (dashed lines show absolute values, while solid lines show relative values). The illumination was the super-continuum source coupled to a tunable filter. A bandwidth of 10 nm was used for each wavelength sample for the transmission-efficiency measurements. The average absolute transmission efficiencies (from 405 nm to 633 nm) are 87%, 87% and 86% for the color-encoded hologram, the photograph hologram and the Macbeth hologram, respectively. Some of the relative efficiencies are higher than 100% indicating that the hologram pattern acts as an anti-reflection coating (right inset in Fig. 4). It is useful to note that the absolute transmission efficiencies can be even higher by applying a properly designed anti-reflection coating on the unpatterned side of the hologram substrate.

In conclusion, we show that broadband transmissive holograms can be enabled by multi-level diffractive optics, which are much simpler to fabricate and offer polarization independence than when compared to alternatives such as those based upon metasurfaces. This is another example of an application where diffractive optics is sufficient when manipulating scalar properties of the electromagnetic field (intensity in this case). Multi-level diffractive optics can be readily manufactured at low cost via embossing techniques^23^ that have been used to create surface-relief-based Bragg holograms. However, unlike Bragg holograms, multi-level diffractive holograms can be transmissive and create images that are relatively invariant with viewing angle. We note that there has been significant progress in the design methods of color computer-generated holograms (CGHs) using techniques like depth division and space division to multiplex the images at different wavelengths^24–26^. However, experimental demonstration of such devices often utilize spatial-light modulators and therefore are constrained by the associated space-bandwidth product. Furthermore, these require relatively narrowband sources such as lasers or light-emitting diodes. Broadband white sources have been used for certain color CGHs, however these suffer from significant chromatic aberations especially at higher resolutions.

## Electronic supplementary material

Supplementary Information
