# Supplementary material for: Full-color, large area, transmissive holograms enabled by multi-level diffractive optics
Source: Sci Rep. 2017 Jul 19;7:5789. doi: 10.1038/s41598-017-06229-5 (PMC5517427; doi:10.1038/s41598-017-06229-5)
Supplement: Supplementary file 1 — Supplementary Information [file 41598_2017_6229_MOESM1_ESM.pdf]

Supplementary Information

Full-color, large area, transmissive holograms enabled by multi-level diffractive optics

Nabil Mohammad[1], Monjurul Meem[1], Xiaowen Wan[1], and Rajesh Menon[1,*]

[1]Department of Electrical and Computer Engineering, University of Utah, Salt Lake City UT 84112

*rmenon@eng.utah.edu

1. Design and geometric parameters of the holograms

The holograms are pixelated in X and Y directions, height of each pixel is quantized into multiple levels. Different parameters have been used for the three hologram designs. These are summarized in Table S1.

Table S1: Design and geometric parameters of three hologram designs.

**Hologram design**	**Color-encoded**	**Macbeth color-chart**	**Photograph (also referred to as Park Building)**
**Pixel size (µm)**	10	20	20
**Number of pixels**	201 X 201	500 X 500	500 X 500
**Physical size (mm X mm)**	2.01 X 2.01	10 X 10	10 X 10
**Maximum height (µm)**	2	2	2.4
**Number of grayscale levels**	8	64	88
**Propagation distance (mm)**	50	500	500
**Design wavelengths (nm)**	405, 532, 633	405, 532, 633	405, 532, 633
**Refractive index at design wavelengths**	1.6894, 1.6482, 1.6347	1.6894, 1.6482, 1.6347	1.6894, 1.6482, 1.6347

The simulated diffraction efficiencies for the three designs are summarized in Table S2 below.

Table S2: Simulated diffraction efficiencies.

**Channel**	**Color-encoded**	**Macbeth color-chart**	**Photograph**
Red(633)	71.33	63.49	77.09
Green(532)	76.77	53.11	84.77
Blue(405)	74.63	65.24	86.30

2. Fabrication

Direct laser-write lithography [1] was used to fabricate the holograms in a single lithography step using the grayscale mode. Shipley 1813 photoresist [2] was spin coated on RCA cleaned 2" D263 glass wafers at 1000 rpm. The samples were then baked in an oven at 110 degree C for 30 minutes. The hologram designs were written on the samples using Heidelberg Micro Pattern Generator 101 tool [3] and developed in AZ 1:1 solution [4] for 1 minute 30 seconds. A calibration step was performed on a separate sample (prepared with the same process conditions) before fabricating the holograms. The goal of calibration step was to determine the exposed depths at a particular gray scale level. Details of the calibration have been discussed elsewhere [5].

3. Imaging setup

We used 3 different methods to characterize the holograms. A color sensor (DFM 22BUCO3-ML, Imaging Source) [8] was placed at the reconstruction plane of the hologram to record the image as shown in Fig.

S1. Secondly, we projected the image onto a translucent white screen and photographed the image from behind but on-axis as illustrated in Fig. S2 (a). Thirdly, we also simply projected the image onto a white screen and photographed the reflected image (Fig. S2 (b)). We used two different illumination sources. One was collimated and expanded beam from SuperK VARIA filter [6] which in turns connected to SuperK EXTREME EXW-6 source [7] while the other was a white collimated backlight. In the case of the color-encoded hologram, the device was illuminated with the three wavelengths from the SuperK (415 nm, 532 nm, 633 nm with 10 nm bandwidth) one by one and corresponding reconstructed images were captured with the setup shown in Fig. S1. These images are shown in Fig. S6 and S7. The images were also projected onto a white screen and photographed using the setup shown in Fig. S2(b). These images were shown in Fig. 1(g-i). Note that 415 nm was used instead of 405 nm because of the low quantum efficiency of the colors sensor at this wavelength and due to the low power from our source at 405nm. In case of the Macbeth color-chart and the photograph holograms, the reconstructed images were captured using the setups shown in Fig. S2. The image shown in Fig. 2(e) was taken using the setup shown in Fig. S2(a) while those in Figs. 2(f), 3(e) and 3(f) were taken with the setup shown in Fig. S2(b). The illumination source was SuperK (405nm-633nm) for the images in Figs. 2(e) and 2(f) and collimated white backlight for those Figs. 3(e) and 3(f). More details about the source spectra are described in the next section.

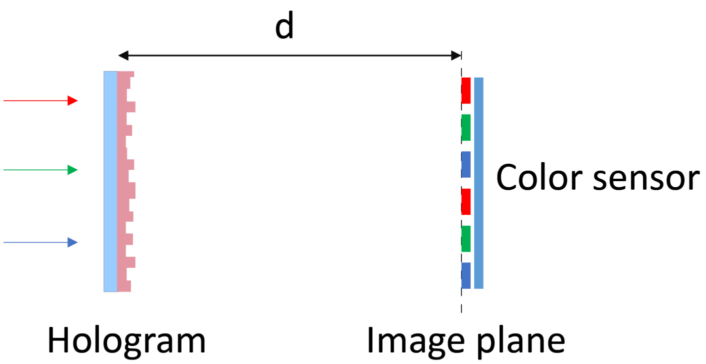

Fig. S1: Schematics showing characterization of the hologram designs using a color sensor.

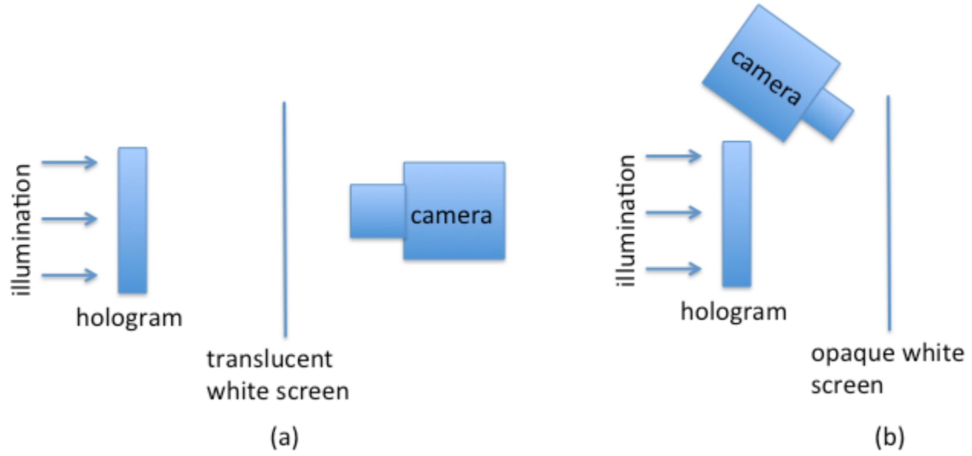

Fig. S2: Schematic showing two other methods for characterizing the image projected by the hologram.

3. Incident spectra

As describe in the last section, we used two types of illumination for the holograms. The first used a super-continuum source (NKT Photonics) coupled with a tunable filter (Varia from NKT Photonics), which allows us to create spatially coherent light, whose central wavelength and bandwidth can be selected. We performed narrowband illumination to characterize the color-encoded holograms as described in the main text. For all other holograms, we used the full visible spectrum and the measured incident spectrum from this source is shown in Fig. S3 (blue curve).

The second source we used was a collimated white backlight placed about 2m away from the hologram to ensure that the illumination was collimated. The incident spectrum of this source is shown in Fig. S3 (red curve).

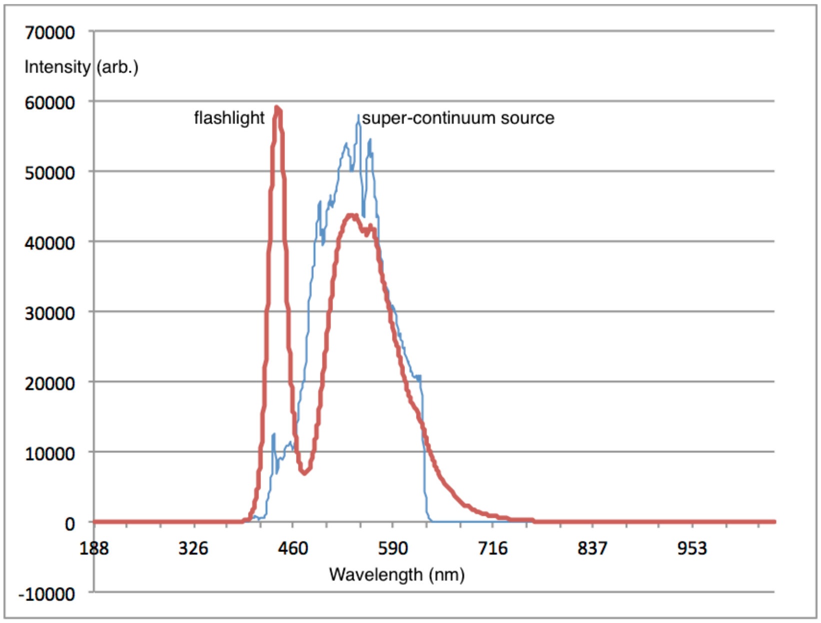

Fig. S3: Measured incident spectra for visible illumination.

4. Design of color-encoded hologram

The color-encoded hologram was designed for λ=405nm, 532nm, 633nm. Due to the low quantum efficiency of the color sensor, illumination wavelength was set to 415nm instead of and this result was shown in the main text. The simulated image at the design wavelength in blue, λ=405nm is shown in Fig. S4. The difference is minimal between 415nm and 405nm.

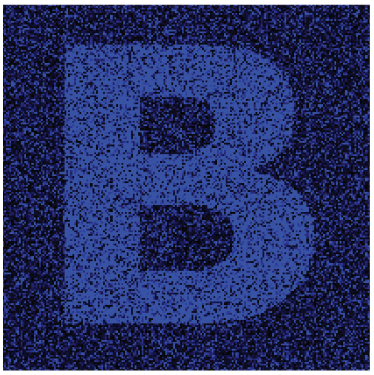

Fig. S4: Simulated image of the color-encoded hologram, when illuminated at λ=405nm.

5. Metrology of the fabricated color-encoded hologram

The pixel heights of the fabricated color-encoded hologram were measured using a stylus profilometer. Five pixel rows were measured for simplicity. The results along with the design values are summarized in Fig. S5. From this data, we estimated an average error in pixel height of 46nm and a standard deviation of 33nm.

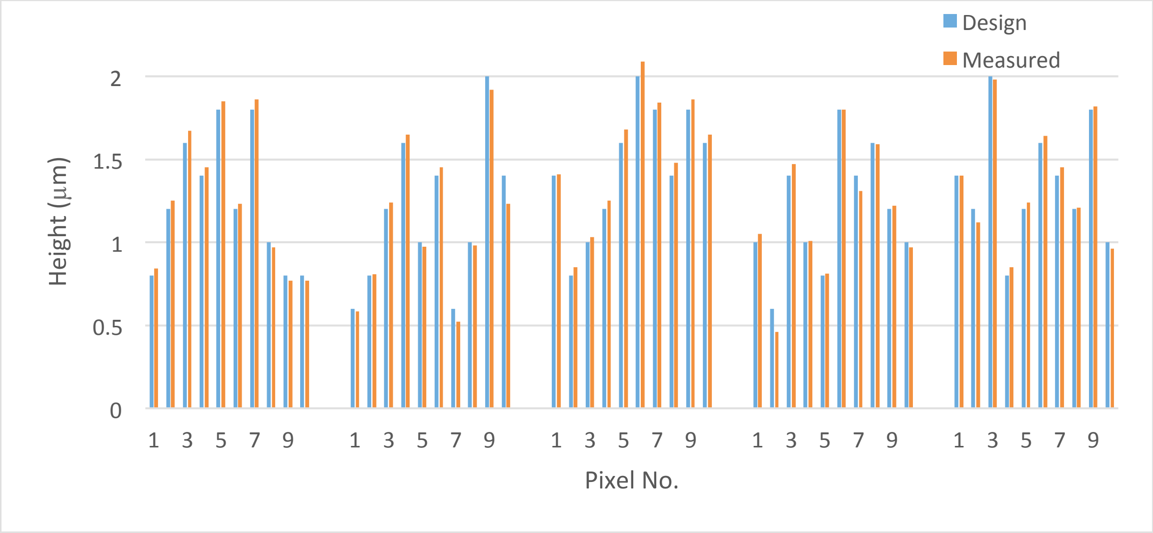

Fig. S5: Measured pixel heights compared to design values for 5 rows of pixels for the color-encoded hologram.

7. Images captured on sensor

In case of the color-encoded hologram, the diffraction efficiencies were determined from the images captured on the color image sensor. The captured images are shown in Fig. S6 below.

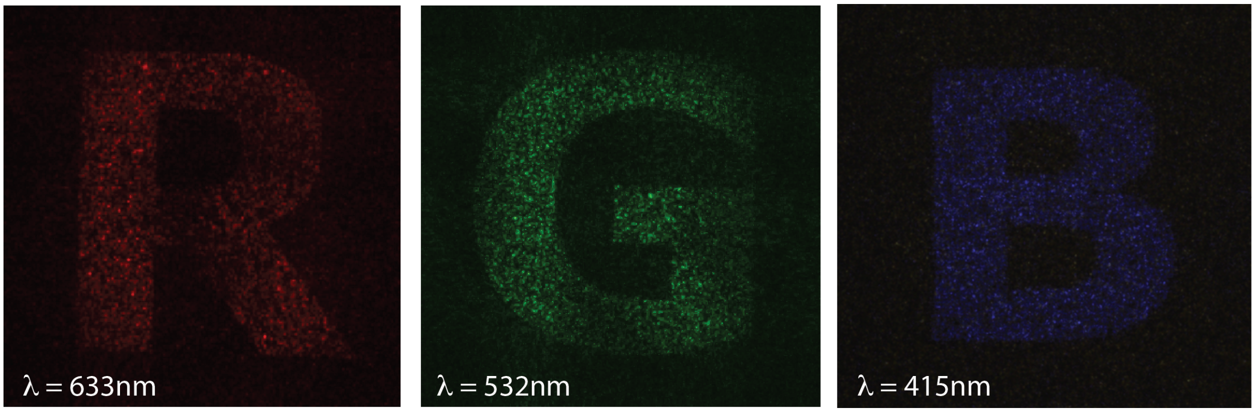

Fig. S6: Images captured on color sensor for the color-encoded hologram.

8. Spectral analysis

The holograms were designed for three discrete wavelengths: 405 nm, 532 nm and 633 nm. To investigate the spectral response of the hologram designs, the holograms were illuminated at 14 different wavelengths with 10 nm bandwidth. Here, the response of only the color-encoded hologram is presented. The setup shown in Fig. S1 was used for this purpose. For each illumination wavelength, corresponding reconstructed image was captured by the color sensor. In each case, a dark image was also recorded and subtracted from the reconstructed images. These raw images were then normalized and converted into gray scale images. Both the raw and grayscale images are presented in Fig. S7.

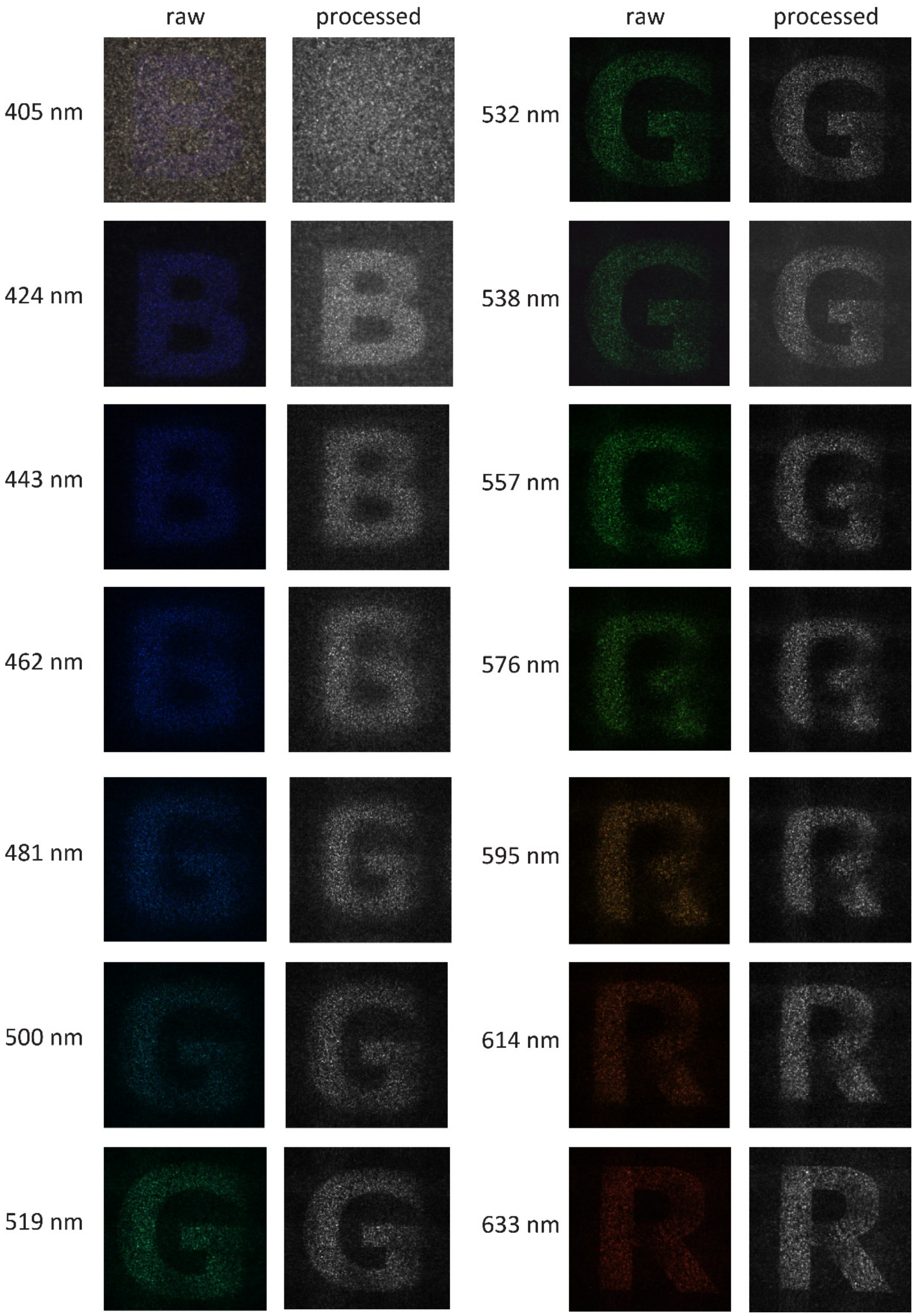

Fig. S7: Experimental images of the color-encoded hologram at different wavelengths.

9. Calculation of transmission and diffraction efficiency

An aperture with dimension equal to one period of the hologram design was placed in front of it (see Fig. S8). A power meter (PM 100A-S130C sensor, Thorlabs) was used to measure the power of the transmitted beam through the hologram pattern, unpatterned photoresist and aperture. Absolute transmission efficiency was then calculated by taking the ratio of the power through the hologram pattern and power through the aperture. Ratio of the power through the hologram pattern and unpatterned photoresist was used to calculate the relative transmission efficiency.

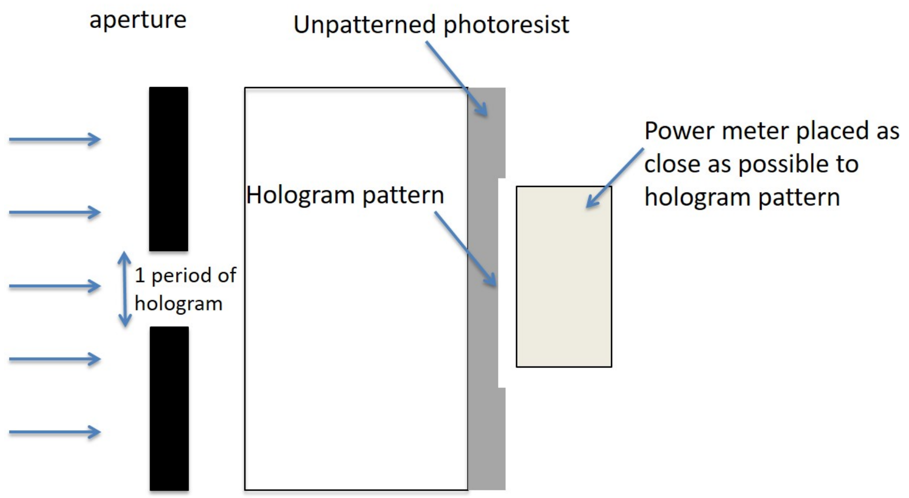

Fig. S8: Setup used to measure the transmission efficiency of the holograms.

To determine the diffraction efficiency of the color-encoded hologram experimentally, the ratio of the intensity integrated over the letters to the intensity over one period was calculated from the images captured by the sensor. Corresponding dark images were subtracted in each case.

10. Design of 1500 X 1500 pixel hologram

As described in the main text, we also designed a high-resolution hologram that projects a photographic image. In this case, we used 1500 X 1500 pixels for one period. The distance between hologram and the image was 1m. The designed height distribution of this device is shown in Fig. S9(a) and 50 X 50 pixels in the top left corner are shown in Fig. S9(b). The simulated image is shown in Fig. S9(c). A photograph of the fabricated device is shown in Fig. S9(d).

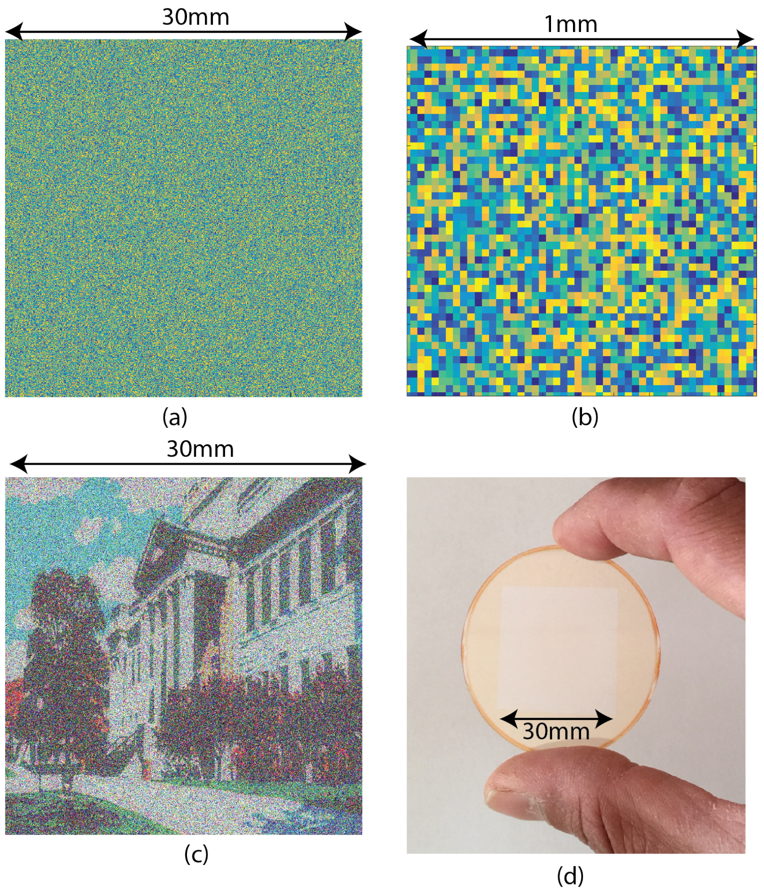

Fig. S9: Hologram of photograph with 1500 X 1500 pixels. (a) Designed height distribution. (b) 50 X 50 pixels in the top left corner. (c) Simulated image. (d) Photograph of fabricated device.

11. Images at various viewing angles

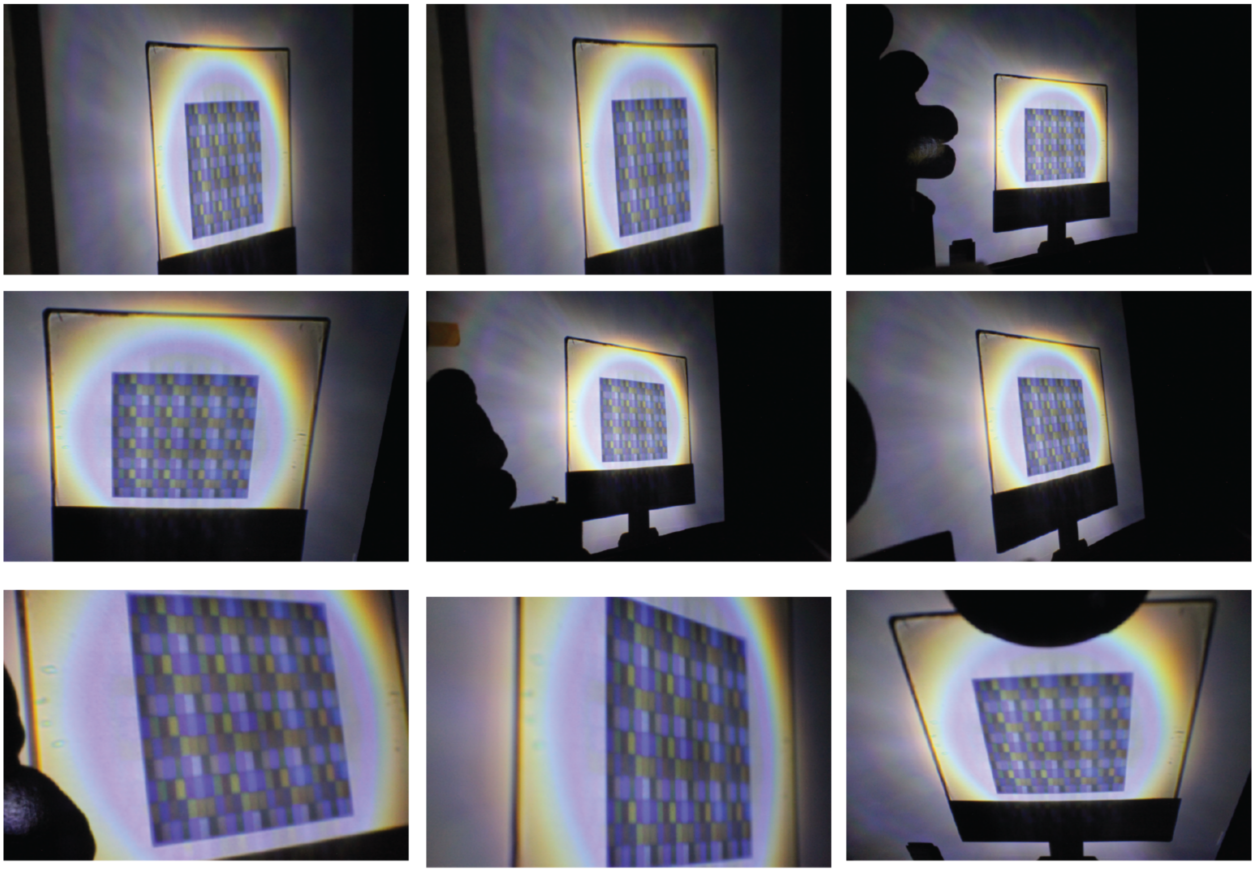

Fig. S10: Images of the Macbeth hologram result at various viewing angles.

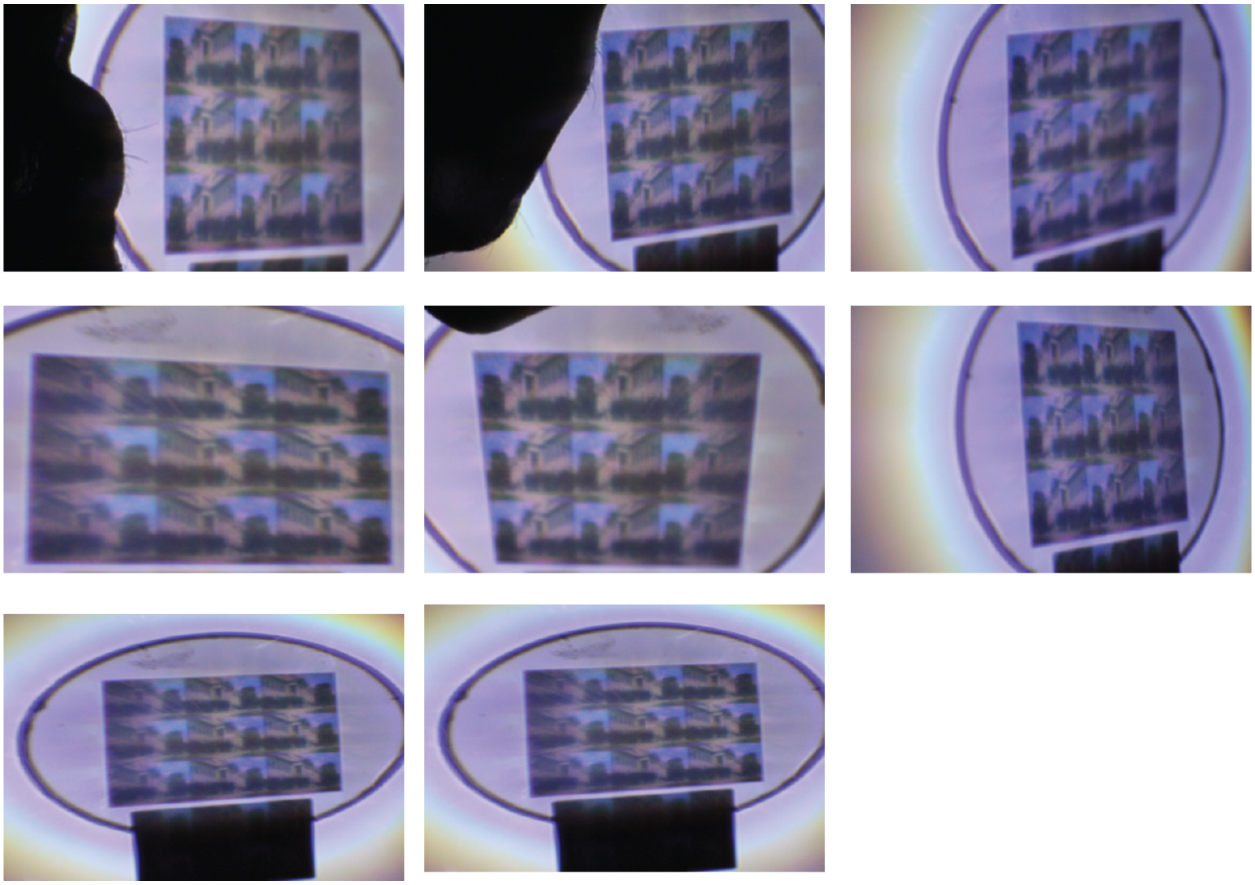

Fig. S11: Images of the color-photograph hologram result at various viewing angles.

References

[1] McKenna, C., Walsh, K., Crain, M. & Lake, J. Maskless Direct Write Grayscale Lithography for MEMS Applications. In *Micro/Nano Symposium (UGIM), 2010 18th Biennial University/Government/Industry*, pp. 1-4. IEEE, 2010.

[2] Data sheet of Shipley 1813 photoresist: http://www.microchem.com/PDFs_Dow/S1800.pdf

[3] Data sheet of Heidelberg μPG 101: http://www.himt.de/index.php/upg-101.html

[4] Data sheet of AZ developer: http://www.microchemicals.com/micro/az_developer.pdf

[5] Wang, P., Dominguez-Caballero, J. A., Friedman, D. J. & Menon., R. A new class of multi-bandgap high-efficiency photovoltaics enabled by broadband diffractive optics. *Prog. Photovolt: Res. Appl.* **23**, 1073-1079 (2015).

[6] Data sheet of SuperK Varia Filter:

http://www.nktphotonics.com/wp-content/uploads/2015/03/SuperK_VARIA.pdf

[7] Data sheet of SuperK Extreme:

http://www.nktphotonics.com/wp-content/uploads/2015/05/superk-extreme.pdf

[8] Data sheet of color camera:

https://s1.dl.theimagingsource.com/api/2.0/packages/publications/factsheets-single/fsdfm22buc03ml/c059478acb/fsdfm22buc03ml.en_US.pdf
